# Humidity-enhanced wet adhesion on insect-inspired fibrillar adhesive pads

**DOI:** 10.1038/ncomms7621

**Published:** 2015-03-20

**Authors:** Longjian Xue, Alexander Kovalev, Anna Eichler-Volf, Martin Steinhart, Stanislav N. Gorb

**Affiliations:** 1 Institut für Chemie neuer Materialien and Zentrum für Physik und Chemie neuer Materialien, Universität Osnabrück, Barbarastrasse 7, 49069 Osnabrück, Germany; 2 Functional Morphology and Biomechanics, Zoological Institute, Kiel University, Am Botanischen Garten 9, 24118 Kiel, Germany

## Abstract

Many insect species reversibly adhere to surfaces by combining contact splitting (contact formation via fibrillar contact elements) and wet adhesion (supply of liquid secretion via pores in the insects’ feet). Here, we fabricate insect-inspired fibrillar pads for wet adhesion containing continuous pore systems through which liquid is supplied to the contact interfaces. Synergistic interaction of capillarity and humidity-induced pad softening increases the pull-off force and the work of adhesion by two orders of magnitude. This increase and the independence of pull-off force on the applied load are caused by the capillarity-supported formation of solid–solid contact between pad and the surface. Solid–solid contact dominates adhesion at high humidity and capillarity at low humidity. At low humidity, the work of adhesion strongly depends on the amount of liquid deposited on the surface and, therefore, on contact duration. These results may pave the way for the design of insect-inspired adhesive pads.

## Abstract (web summary)

Many insects supply secretion via small tubes or pores to the end of their legs to be able to attach to a surface. Here, inspired by nature, Xue *et al.* fabricate adhesive pads with porous nanorod structure for oil delivery, which give rise to a 100-fold increase in adhesivity under humid conditions.

## Graphical abstract

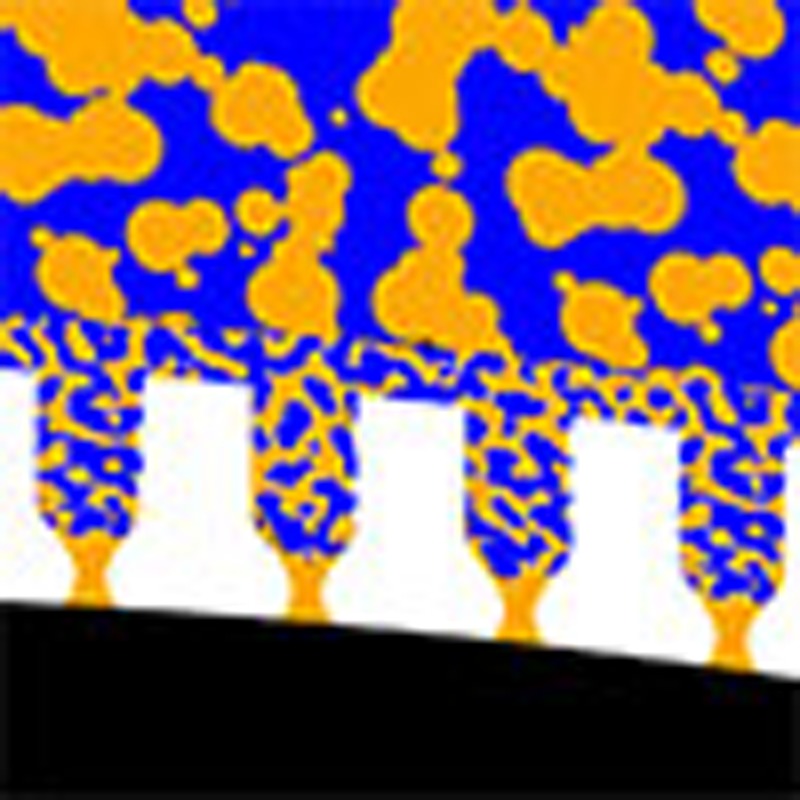

Micro- and nano-scaled fibrillar adhesive pads based on the contact splitting principle may show adhesive strengths matching or even surpass those of natural systems^1^,^2^. The contact splitting principle involves the contact formation between the discrete fibrillar contact elements of an adhesive pad and a counterpart surface at a large number of discrete contact points. Thus, fibrillar adhesive pads exhibit high adaptability to rough or curved counterpart surfaces, constrained crack propagation, pronounced adhesion reversibility and high durability. While bioinspired fibrillar adhesive pads have predominantly been designed for dry adhesion based on van der Waals forces mimicking gecko adhesion^3^,^4^, nature has developed elegant solutions to further optimize the performance of biological fibrillar adhesive pads. Softening of fibrillar adhesive pads by the presence of hydrated biopolymers increases their mechanical compliance, their adaptability to rough counterpart surfaces and ultimately their adhesive strength. For example, the hydration of resilin drastically decreases the Young’s modulus in the setal tips of the beetle *Coccinella septempunctata* from 7.2 GPa to 1.2 MPa, resulting in a significantly improved adhesion performance^5^. Furthermore, enhanced compliance of fibrillar adhesive pads by hydration has been considered as the reason for the pronounced dependence of dry gecko adhesion on relative humidity (RH)^6^. On the other hand, wet adhesion of insects^7^,^8^,^9^,^10^,^11^,^12^ is caused by capillary forces related to the presence of adhesive secretions at the contact interface. Direct solid–solid interactions between cuticle pad and counterpart surface, as well as capillary contributions related to the presence of adhesive liquids at the contact interface, were reported to concurrently contribute to the adhesion of Asian weaver ants (*Oecophylla smaragdina*)^9^. The adhesive secretions are transported to the contact interfaces through channels or sponge-like pore systems inside the insects’ setae^13^,^14^. Secretion footprints remaining after the contact between insect feet and counterpart surfaces have been related to mechanically controlled, continuous supply of secretion from the interior of insects’ setae on the feet to the contact interface^15^,^16^. Despite the intriguing design principles biological models use to supply adhesive secretions to contact interfaces, little efforts have been directed to the design of artificial fibrillar adhesive pads exploiting wet adhesion. One of the few examples reported to date enables adhesion switching by pumping water forth and back through micromachined arrays of holes with diameters of several tens of microns^17^.

In this work, we transfer a combination of wet adhesion and hydration-induced softening to bioinspired porous fibrillar adhesive pads (PFAPs). PFAPs consist of arrays of block copolymer nanorods serving as fibrillar contact elements, which are supported by a film-like substrate consisting of the same material. Most importantly, PFAPs contain continuous sponge-like pore systems open to the environment that enable the delivery of liquid from the smooth underside of the PFAP substrate to the tips of the fibrillar contact elements. As shown below, synergistic interaction of humidity-induced PFAP softening and wet adhesion via liquids supplied from PFAP undersides results in capillarity-supported formation of solid–solid contact between the liquid-infused PFAP and the counterpart surface (Fig. 1), resulting in adhesion enhancement and increase in the work of adhesion by two orders of magnitude. Capillarity-supported formation of solid–solid contact also results in the independence of pull-off force and work of adhesion from the applied load. At low RH, capillarity contributions (which are independent on RH) dominate the work of adhesion; the more the liquid is deposited on the counterpart surface, the higher are the work of adhesion and rupture distances of liquid bridges between the liquid-infused PFAP and the counterpart surface.

## Results

### Supply of liquid to the contact interface

The PFAPs (Fig. 2a,b) with an overall thickness of ≈110 μm consisted of the block copolymer polystyrene-*block*-poly(vinyl-2-pyridine) (PS-*b*-P2VP) and were prepared as described elsewhere^18^. The hydrophobic component PS formed a rigid, glassy skeleton, whereas the internal pore walls, as well as the outer surfaces, consisted of the polar component P2VP. The fibrillar contact elements (diameter≈300 nm; length≈1.5 μm) with hemispherical tips formed an array with a nearest-neighbour distance of≈500 nm. The average diameter of the internal pores of the PFAPs amounted to≈98 nm. The permeability of PFAPs was tested with mineral oil, which we selected as model for the oily secretions of insect setae^19^. The contact angles of mineral oil on P2VP and the spherical sapphire probe used for adhesion tests (see below) are summarized in the Supplementary Table 1. Imbibition tests with mineral oil revealed that the imbibition front propagated with an initial velocity of≈180 μm s^−1^ in the substrate planes of the tested PFAPs, whereas propagation slowed down after travel distances in the millimetre range (Fig. 2c). This outcome is in qualitative accordance with a Lucas–Washburn-type imbibition kinetics^20^. We estimate that mineral oil supplied from the smooth underside of a ~110 μm-thick PFAP reaches the tips of the fibrillar contact elements in <600 ms.

To demonstrate liquid delivery from the smooth undersides of PFAPs to the tips of the fibrillar contact elements, the smooth underside of a PFAP was placed on tissue paper (Fig. 3a). Mineral oil (typically 10 μl) was then dropped on the tissue paper in such a way that the tissue paper and the continuous pore system of the tested PFAP were just saturated, while submersion of the fibrillar contact elements was avoided (Fig. 3b). Mineral oil-infused PFAPs will thereafter be referred to as oPFAPs. In a typical experiment, the fibrillar contact elements of an oPFAP were carefully brought into contact with a glass slide and then detached. As revealed by white-light interferometry, discrete oil droplets with diameters ranging from ~300 nm to ~2.5 μm and heights of several tens of nanometres were deposited on the glass slide (Fig. 3c). The sizes of the mineral oil droplets were commensurate with the diameters of single fibrillar contact elements or small bunches of them. It is reasonable to assume that mineral oil bridges formed between single fibrillar contact element or small clusters of fibrillar contact elements and the counterpart surface, and ruptured upon detachment thus generating the droplet pattern seen in Fig. 3c.

### Differences between wet adhesion and dry adhesion

Adhesion on PFAPs at RH≈25% was measured using a previously described method^18^,^21^ at room temperature. In brief, force-displacement curves were obtained by employing spherical sapphire probes with a diameter of 3 mm mounted on metal springs. A force-displacement curve measured on a PFAP in the absence of liquid at the contact interface representing a typical dry adhesion scenario is shown in Fig. 4a. For comparison, Fig. 4b shows a force-displacement curve measured on an oPFAP with a spherical sapphire probe already contaminated with mineral oil representing a typical wet adhesion scenario. In the approach branch of the force–distance curve (Fig. 4a) detected force equalled to zero until solid–solid contact between the spherical sapphire probe and the tested PFAP was formed. The solid–solid contact is assumed to form as soon as a positive force has to be applied to further displace the spherical sapphire probe into the tested PFAP. After the formation of solid–solid contact, the spherical sapphire probe was further displaced into the PFAP until a predefined positive loading force *F*_L_ was reached. In contrast to the approach part seen in Fig. 4a, the approach part seen in Fig. 4b shows a marked jump-in event before the formation of the solid–solid contact. The jump-in associated with an attractive (that is, negative) jump-in force *F*_J_ started at the jump-in distance *D*_J_. *D*_J_ corresponds to the displacement of the spherical sapphire probe from the position where the jump-in started to the position where the solid–solid contact is formed. The jump-in events, observed during the approach of mineral oil-contaminated spherical sapphire probes to the oPFAPs, were reminiscent of the jump-in events commonly observed during the approach of atomic force microscopy cantilever tips to counterpart surfaces caused by capillary condensation of water bridges^22^. We observed no jump-in during the first attach/detach cycle after mineral oil supply (for example, attach/detach cycle 1 in Fig. 5a), because the surface of the spherical sapphire probe was not contaminated with mineral oil during approach. However, the formation of solid–solid contact in the course of the first attach/detach cycle resulted in the transfer of mineral oil to the surface of the spherical sapphire probe. Thus, jump-in occurred in the second attach/detach cycle after mineral oil supply (for example, attach/detach cycle 2 in Fig. 5a) and in any further attach/detach cycle (attach/detach cycles 3–6 in Fig. 5a). Humidity can be ruled out as the origin of jump-in, since no indications of jump-in were apparent in the control experiments conducted in the absence of mineral oil within a RH range from 25 to 90% (ref. 18).

The retraction branch of the force-displacement curve seen in Fig. 4a starts as soon as the force maximum corresponding to *F*_L_ has been passed. The applied force decreases during retraction and passes the zero-force line. Beyond the intersection of the retraction branch and the zero-force line, adhesion to the spherical sapphire probe drags the tested PFAP out of its equilibrium position, and the measured force becomes negative. Eventually, the retraction branch passes a sharp force minimum that is immediately followed by a sharp increase in force to the zero-force line. This shape of the retraction branch indicates instantaneous solid–solid separation at the displacement corresponding to the force minimum. The force minimum corresponds to the pull-off force (or adhesion force) *F*_ad_ required to detach the spherical sapphire probe from the tested PFAP. In the presence of mineral oil at the contact interface (Fig. 4b), the retraction branch of the force-displacement curve also shows a distinct force minimum corresponding to *F*_ad_. However, after having passed the force minimum, the force–distance curve does not instantaneously relax to the zero-force line. Instead, it slowly approaches the zero-force line as the spherical sapphire probe is further retracted beyond the position of the force minimum. This feature of force-displacement curves measured in the presence of mineral oil at the contact interface can be attributed to the stretching and rupturing of liquid bridges formed between the oPFAP and the spherical sapphire probe after the separation of the solid–solid contact. The apparent rupture distance *D*_R_ corresponds to the distance between the position of the force minimum *F*_ad_ and the position beyond which the retraction branch coincides with the zero-force line. Thus, in the presence of mineral oil at the contact interface the force–distance curve shows pronounced hysteresis similar to atomic force microscopy force–distance curves measured with blunt cantilever tips^23^.

### Wet adhesion at RH=25%

Figure 5a shows *D*_J_, *F*_J_, *F*_ad_ and *D*_R_ values obtained from a series of force–distance curves successively measured at the same position on a PFAP/oPFAP before and after the supply of mineral oil at *F*_L_≈500 μN and RH=25%. Supply of mineral oil resulted in an increase in *F*_ad_ by one order of magnitude from 3.1±1.9 μN (average of three measurements at different positions on the tested PFAP) before mineral oil supply to 74.5 μN in the first attach/detach cycle after mineral oil supply (attach/detach cycle 1 in Fig. 5a). Further attach/detach cycles carried out at the same location yielded slightly decreased *F*_ad_ values of≈56.7 μN that remained by and large constant over five further attach/detach cycles. *F*_J_ apparent in attach/detach cycles 2–6 after supply of mineral oil amounted to≈52.8 μN and remained by and large constant too. It is interesting to note that *F*_ad_ approximately equalled *F*_J_. *D*_J_ increased from 0.9 μm in the second attach/detach cycle after mineral oil supply (first occurrence of a jump-in) to 1.9 μm in the third attach/detach cycle and leveled off at ~3.0 μm in the attach/detach cycles subsequently measured at the same location. *D*_R_ continuously increased from 4.8 μm in the first attach/detach cycle after mineral oil supply to≈18 μm. To investigate the dependence of *F*_ad_ and *D*_R_ on *F*_L_, we measured a series of force-displacement curves with successively increased *F*_L_ at the same position on an oPFAP. Before the *F*_L_-dependent adhesion measurements, the spherical sapphire probe had 13 contacts with the same oPFAP at different positions. *F*_ad_ and *D*_R_ are largely independent of *F*_L_ in the *F*_L_ range from 45 to 800 μN (Fig. 5b). The *F*_ad_ value of ~56 μN apparent from Fig. 5b corresponded to the *F*_ad_ value obtained in the six successive attach/detach cycles measured at *F*_L_=500 μN seen in Fig. 5a. Moreover, Fig. 5b reveals that *D*_R_ leveled off at ~22 μm. This outcome indicates that at RH=25% *D*_R_ does not depend on *F*_L_. Moreover, the spherical sapphire probe was evidently saturated with mineral oil after the 13 previous contacts with the oPFAP so that during each of the measurements displayed in Fig. 5b liquid bridge rupture during retraction occurred in the same way.

### Wet adhesion at RH=90%

While at RH=25% the effective elastic modulus *E*_eff_ of PFAPs amounted to 25.0 MPa, exposure of PFAPs to RH=90% for 4 min reduced *E*_eff_ to 4.2 MPa (Supplementary Fig. 1). Because of their enhanced mechanical compliance at RH=90%, PFAPs exhibit significantly better adhesive performance than at RH=25% (ref. 18). Supply of mineral oil at RH=90% led to an additional sharp jump in *F*_ad_ by 389 μN from 166.3±25.4 μN (average of three measurements at different positions on the tested PFAP) to 569 μN for *F*_L_≈390 μN (Fig. 5c). In the five subsequent attach/detach cycles, *F*_ad_ leveled off at≈530 μN. Similar to the results obtained for wet adhesion at RH=25%, at RH=90% the *D*_R_ values were larger than the *D*_J_ values, and both the *D*_R_ and *D*_J_ values increased in successively performed attach/detach cycles. At RH=90% *F*_J_ also increased during attach/detach cycles 2–6 after the supply of mineral oil but remained much smaller than *F*_ad_ (Fig. 5c). The dependence of *F*_ad_ and *D*_R_ on *F*_L_ is shown in Fig. 5d. Before the *F*_L_-dependent measurements, the sapphire sphere had 10 contacts with the tested oPFAP at a different position and was saturated with mineral oil. Such as at RH=25%, at RH=90% *F*_ad_ was independent of *F*_L_ within the tested *F*_L_ range. In the absence of liquid at the contact interface, *F*_ad_ at RH=90% monotonically increased along with *F*_L_ within the considered *F*_L_ range from *F*_ad_=16.2 μN for *F*_L_=39.4 μN to *F*_ad_=135.2 μN for *F*_L_=552.7 μN (Supplementary Fig. 2). Likewise, *D*_R_ had a constant value of ~35 μm independent of *F*_L_.

### Work of adhesion

The work of adhesion *W*_ad_ is defined as the work required to separate the contacting surfaces. *W*_ad_ corresponds to the area enclosed by the retraction branch of a force-displacement curve and the zero-force line between the intersection retraction branch/zero-force line and the displacement beyond which the retraction branch coincides with the zero-force line (Fig. 6). *W*_ad_ can be separated into a contribution *W*_ad,S_ required to break the solid–solid contact between fibrillar contact elements and spherical sapphire probe and a capillary contribution *W*_ad,C_ required for stretching and rupturing the liquid bridges between the oPFAP and the spherical sapphire probe. *W*_ad,S_ corresponds to the area enclosed by the retraction branch and the zero-force line between the intersection retraction branch/zero-force line and the displacement corresponding to the force minimum *F*_ad_ (area marked by dark blue lines in Fig. 6). *W*_ad,C_ corresponds to the area enclosed by the retraction branch and the zero-force line between the displacement corresponding to the force minimum *F*_ad_ and the displacement beyond which the retraction branch coincides with the zero-force line (area marked by lines in magenta in Fig. 6).

Delivery of mineral oil to the contact interface resulted in a sharp increase in *W*_ad_. *W*_ad_ at RH=25% and *F*_L_≈500 μN (*W*_ad,25%_) measured in the first attach/detach cycle after mineral oil supply amounted to 1.2 × 10^−10^ J and was 24.5 times larger than *W*_ad,25%_ before mineral oil supply at the same *F*_L_ value. Such as *F*_ad_, *W*_ad,S_ remained by and large constant in attach/detach cycles 1–6 after the supply of mineral oil, even though the *W*_ad,S_ values scattered to some extent from 6.4 × 10^−11^ J to 2.9 × 10^−11^ J (Fig. 7a). *W*_ad,C_ monotonically increased in the six attach/detach cycles successively measured after mineral oil supply from 5.5 × 10^−11^ J (first attach/detach cycle after mineral oil supply) to 2.6 × 10^−10^ J (sixth attach/detach cycle after mineral oil supply). In the second attach/detach cycle after mineral oil supply and in all further cycles, *W*_ad,C_ was by far the dominant contribution to *W*_ad,25%_. After >10 contacts of spherical sapphire probe and oPFAP, the sapphire surface was apparently saturated with mineral oil; *W*_ad,C_ leveled off at ~4 × 10^−10^ J and *W*_ad,S_ at 4 × 10^−11^ J. Once *W*_ad,S_ and *W*_ad,C_ had reached these values, *W*_ad,S_ and *W*_ad,C_ were evidently independent of *F*_L_ (Fig. 7b).

*W*_ad_ at RH=90% (*W*_ad,90%_) was 8.5 times larger in the presence than in the absence of mineral oil. In the first attach/detach cycle after delivery of mineral oil, *W*_ad,90%_ amounted to 22.7 × 10^−10^ J (Fig. 7c) and was almost 20 times larger than *W*_ad,25%_=1.2 × 10^−10^ J. The difference between *W*_ad,25%_ and *W*_ad,90%_ was nearly exclusively caused by the difference in *W*_ad,S_. *W*_ad,S_ at RH=90% and *F*_L_≈390 μN lay in the range from 19.3 × 10^−10^ J to 23.3 × 10^−10^ J (Fig. 7c) and was, therefore, nearly one order of magnitude larger than at RH=25% and *F*_L_≈500 μN. In contrast to the situation at RH=25%, in all attach/detach cycles at RH=90% *W*_ad,S_ was by far the dominating contribution to *W*_ad,90%_. In the first two attach/detach cycles after supply to mineral oil, the contribution of *W*_ad,C_ to *W*_ad,90%_ was negligible. In attach/detach cycles 3–6 after mineral oil supply, a slight increase in *W*_ad,C_ to 5.1 × 10^−10^ J occurred. After >10 contacts of spherical sapphire probe and oPFAP, *W*_ad,S_ levelled off at ~18 × 10^−10^ J and *W*_ad,C_ at 5 × 10^−11^ J. Again, *W*_ad,S_ and *W*_ad,C_ were independent of *F*_L_ once *W*_ad,S_ and *W*_ad,C_ had reached these values (Fig. 7d).

In contrast to the significantly different *W*_ad,S_ values at RH values of 25 and 90%, the *W*_ad,C_ values obtained at these two RHs lay in a similar range, as obvious from Fig. 7b,d. Therefore, the capillary contribution *W*_ad,C_ to *W*_ad_ is, if at all, only weakly dependent on RH. It is, however, obvious that *W*_ad,C_ correlates with *D*_R_; the larger *D*_R_ is, the larger is *W*_ad,C_ (Fig. 8). Once *D*_R_ remains constant, *W*_ad,C_ also remains constant (Figs 5b and 7b and Figs. 5d and 7d, respectively, show the same set of measurements).

## Discussion

The capillarity contribution to adhesion is caused by the formation of liquid bridges after the separation of the solid–solid contact between the spherical sapphire probe and the oPFAP at the position of the force minimum *F*_ad_. Further retraction leads to the stretching and eventually to the rupture of the liquid bridges. *D*_R_ can be interpreted as the distance between the position of solid–solid contact separation and the position at which all the liquid bridges are ruptured. Consequently, *W*_ad,C_ is the work required to stretch and rupture all the liquid bridges. In contrast to *W*_ad,S_, *W*_ad,C_ does apparently not depend on RH; the magnitude of *W*_ad,C_ was the same for RH values of 25 and 90% (Fig. 8). The implications of this outcome are as follows. (i) *W*_ad,25%_ at RH=25% is dominated by the capillarity contribution *W*_ad,C_, whereas *W*_ad,90%_ at RH=90% is dominated by the solid–solid contribution *W*_ad,S_. (ii) The properties of the liquid bridges formed between the spherical sapphire probe and the oPFAP are not influenced by RH as such or by the humidity-induced softening of the oPFAP induced by an increase in RH. This finding may be rationalized as follows. Before the emergence of liquid bridges, the oPFAPs mechanically relax. Thus, at the moment of liquid bridge formation the actual contact area between the spherical sapphire probe and the oPFAP is similar for the tested RHs. This view is supported by the finding that independent of RH, neither *D*_R_ (Fig. 5b,d) nor *W*_ad,C_ (Fig. 7b,d) depends on *F*_L_ in the tested *F*_L_ range from 45 to 800 μN. Hence, *F*_L_ does not influence liquid bridge formation and rupture. (iii) We can rule out that capillary condensation of water from the gas phase significantly influences adhesion.

Before the first contact between the spherical sapphire probe and the oPFAP after mineral oil supply, the spherical sapphire probe is not contaminated with mineral oil. During retraction in the course of the first attach/detach cycle after mineral oil supply, liquid bridges between single fibrillar contact elements or small bunches of fibrillar contact elements and the spherical sapphire probe form once the solid–solid contact is separated. Hence, patterns of discrete mineral oil droplets remain on counterpart surfaces after rupture of the liquid bridges (see Fig. 3c). Because the spherical sapphire probe has a much smaller curvature than the tips of the fibrillar contact elements, the larger portion of the mineral oil forming the liquid bridges will remain on the spherical sapphire probe when the liquid bridges rupture^24^,^25^. In the first attach/detach cycle after supply of mineral oil and in attach/detach cycles carried out thereafter the rupture of the liquid bridges will redistribute liquid from the oPFAP to the spherical sapphire probe. Hence, the amount of mineral oil on the surface of the spherical sapphire probe increases after every attach/detach cycle. The amount of mineral oil that can be supplied from the surface of the spherical sapphire probe to the formation of the liquid bridges in turn increases in each successively performed attach/detach cycle.

*D*_R_ increases along with the volumes of the rupturing liquid bridges^23^,^26^,^27^,^28^. In attach/detach cycles 1–6 after the supply of mineral oil, *D*_R_ gradually increases for both the tested RH values of 25 and 90%. It is reasonable to assume that the volumes of the liquid bridges formed between the oPFAP and the spherical sapphire probe increase along with the amount of mineral oil that can be supplied to the liquid bridges from the surface of the spherical sapphire probe. Hence, the observed increase in *D*_R_ in attach/detach cycles 1–6 after mineral oil supply (Fig. 5a,c) is presumably related to the ability of the spherical sapphire probe to supply more mineral oil to the liquid bridges as the more mineral oil is available on its surface the more attach/detach cycles were performed. Therefore, *D*_R_ depends on the distribution of liquid between the oPFAP and the spherical sapphire probe; the more mineral oil redistributed to the spherical sapphire probe, the higher is the apparent *D*_R_ value. *W*_ad,C_ increases along with *D*_R_ (Fig. 8). Hence, *W*_ad,C_ gradually increases in attach/detach cycles 1–6 after mineral oil supply (Fig. 7a,c). After about six attach/detach cycles, *D*_R_ (Fig. 5b,d) as well as *W*_ad,C_ (Fig. 7b,d) reach a plateau and remain constant in further attach/detach cycles. Apparently, the liquid distribution between the oPFAP and the spherical sapphire sphere reaches a state of saturation. Either the liquid is no longer redistributed or further liquid redistribution does not influence the structure and the rupture of the liquid bridges. The apparent *D*_R_ values of ~22 μm at RH=25% and of ~35 μm at RH=90% in the saturated state indicate that the liquid bridges are no longer formed between single contact elements or small bunches of contact elements of the oPFAP and the spherical sapphire probe. Instead, the oPFAP apparently immerses into the mineral oil layer on the spherical sapphire probe so that one single liquid bridge between the oPFAP and the spherical sapphire probe is formed.

The contribution of solid–solid contact is represented by *F*_ad_ and *W*_ad,S_. In the retraction branches, *F*_ad_ marks the position at which solid–solid contact is separated, and *W*_ad,S_ is the work required to separate the solid–solid contact. The increases in *F*_ad_ by humidity-induced softening and by the supply of mineral oil to the contact interface are synergistic, resulting in an increase in *F*_ad_ by two orders of magnitude. The increase in *F*_ad_ caused by mineral oil supply at the given RH is evidently larger at RH=90% (increase in *F*_ad_=389 μN; Fig. 5c) than at RH=25% (increase in *F*_ad_=70 μN; Fig. 5a). We assume that this outcome can be rationalized by the larger actual solid–solid contact area at higher RH caused by humidity-induced softening of the PFAPs/oPFAPs. This view is supported by the fact that the presence of mineral oil at the contact interface *W*_ad,S_ increases by nearly one order of magnitude when RH is increased from 25 to 90% (Fig. 7).

In the case of oPFAPs, both *F*_ad_ (Fig. 5a,c) and *W*_ad,S_ (Fig. 7a,c) are independent of the amount of liquid deposited on the spherical sapphire probe. In contrast to *D*_R_ and *W*_ad,C_, both the parameters remain by and large constant in the attach/detach cycles 1–6 performed after the supply of mineral oil as well as after the saturation of the spherical sapphire probe with mineral oil. While *D*_R_ no longer reflects contact splitting if the spherical sapphire probe is saturated with mineral oil, it appears that the solid–solid contact is still dominated by the contact between the single contact elements (or small clusters of contact elements) and the spherical sapphire probe.

The independence of *F*_ad_ on *F*_L_ is a striking difference between wet adhesion on oPFAPs and dry adhesion on PFAPs. Under dry adhesion conditions, *F*_ad_ increases along with *F*_L_ until a plateau is reached. This behaviour could be reproduced with a modified Schargott−Popov−Gorb (SPG) model^18^,^21^. The modified SPG model introduces an additional parameter, the probability of the contact formation between the contact elements and the spherical sapphire probe as a function of *F*_L_. Thus, only a *F*_L_-dependent fraction of contact elements within the contact area is in contact with the spherical sapphire probe. *F*_ad_ depends on the fraction of the contact elements in contact with the spherical sapphire probe and, therefore, on *F*_L_. Only above a certain *F*_L_ threshold value, all the contact elements are in contact with the spherical sapphire probe and *F*_ad_ does not change if *F*_L_ is further increased.

**Figure 1:**
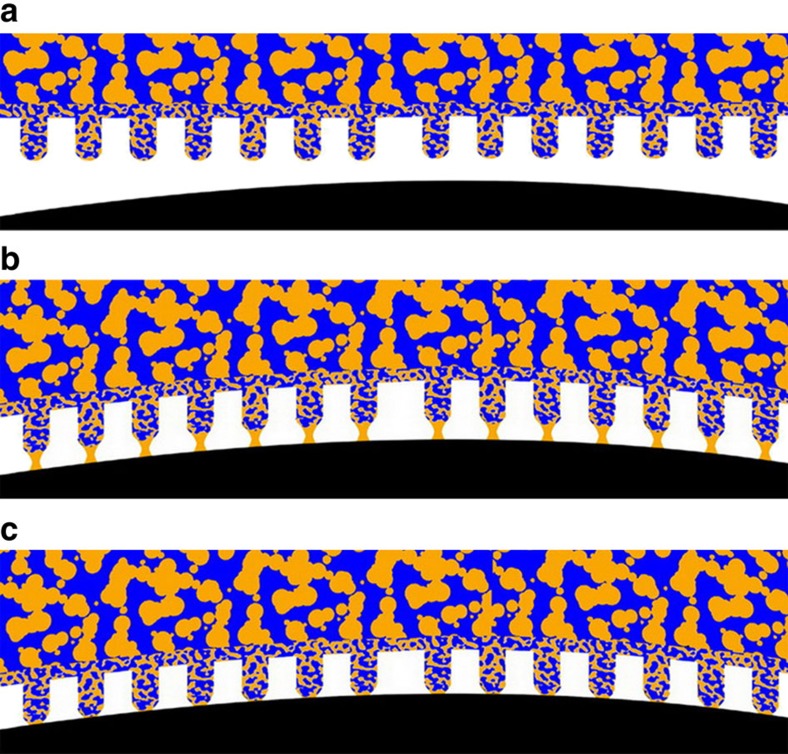
Capillarity-supported formation of solid–solid contact. (**a**) An oPFAP is approached to a counterpart surface such as a spherical sapphire probe. (**b**) Liquid bridges form between the contact elements of the oPFAP and the counterpart surface. (**c**) Liquid bridge-mediated contact formation supports the formation of solid–solid contact between the contact elements and the counterpart surface. The modified SPG model predicts the imperfect contact formation between the contact elements and the counterpart surface if liquid is absent at the contact interface. This imperfect contact formation in turn results in poorer adhesion, as experimentally observed. The colours of black, blue and orange represent sapphire probe, oPFAP (small pores) in contact with tissue paper (big pores) and mineral oil, respectively.

**Figure 2:**
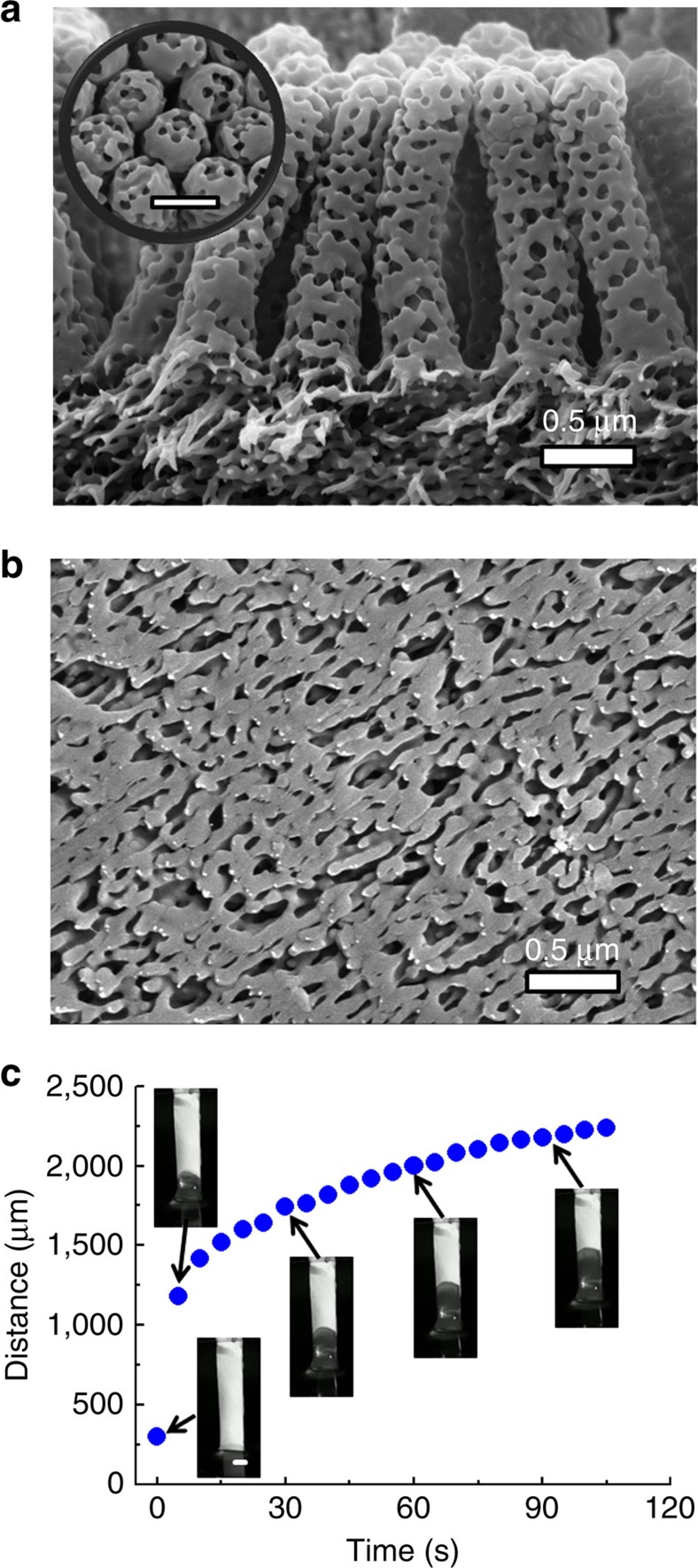
Porous fibrillar PS-*b*-P2VP adhesive pads. Scanning electron microscopy (SEM) images of (**a**) contact elements and (**b**) the smooth substrate underside of a PFAP. The inset in **a** shows tips of contact elements. (**c**) Imbibition of a PFAP with mineral oil. The imbibition kinetics is represented by the travel distance of the imbibition front in the plane of the substrate (normal to the long axes of the contact elements) as a function of the imbibition time. The photographs (scale bar, 1 mm) show the propagation of the imbibition front. The initially opaque PFAP became semitransparent when imbibed with mineral oil.

**Figure 3:**
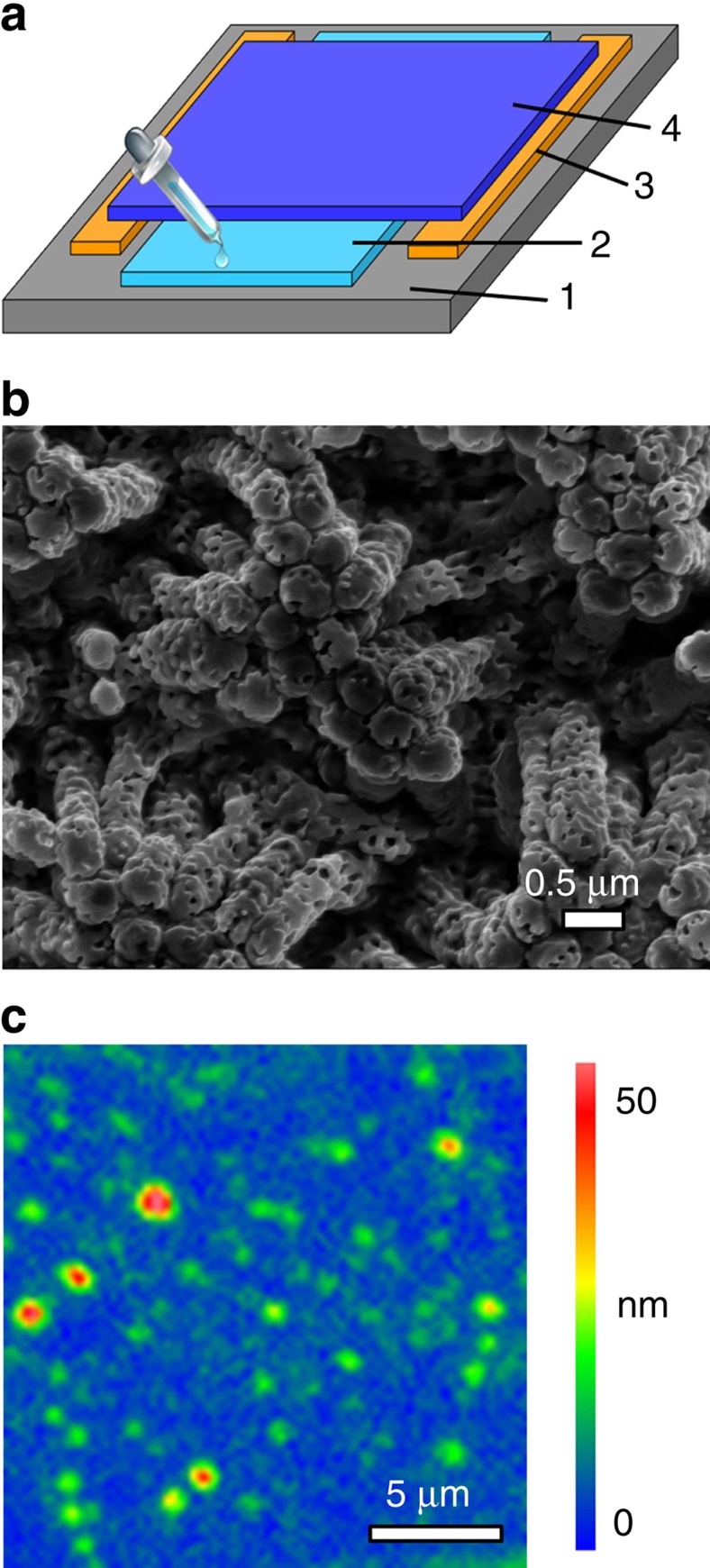
Transfer of mineral oil to a counterpart surface. Mineral oil is supplied via the smooth underside of an oPFAP to the porous nanorods serving as contact elements. (**a**) Diagram of the experimental configuration used for permeation tests. The smooth underside of oPFAP 4 is placed on tissue 2, which is in turn placed on glass slide 1. The contact elements of oPFAP 4 point upwards. oPFAP 4 is fixated on glass slide 1 with adhesive tape 3. Mineral oil is dropped on a portion of tissue 2 not covered by oPFAP 4. The amount of supplied mineral oil is adjusted in such a way that tissue 2 is just saturated with but not submerged in mineral oil. (**b**) Cryo-SEM image of an oPFAP saturated with mineral oil. (**c**) White-light interferometry image showing mineral oil droplets on a glass slide that was gently brought into contact with the tips of the contact elements of an oPFAP. The colour bar encodes the heights of the droplets. The oPFAP, as shown in **b**, used to deposit the mineral oil droplets displayed in **c** were saturated with mineral oil using the configuration shown in **a**.

**Figure 4:**
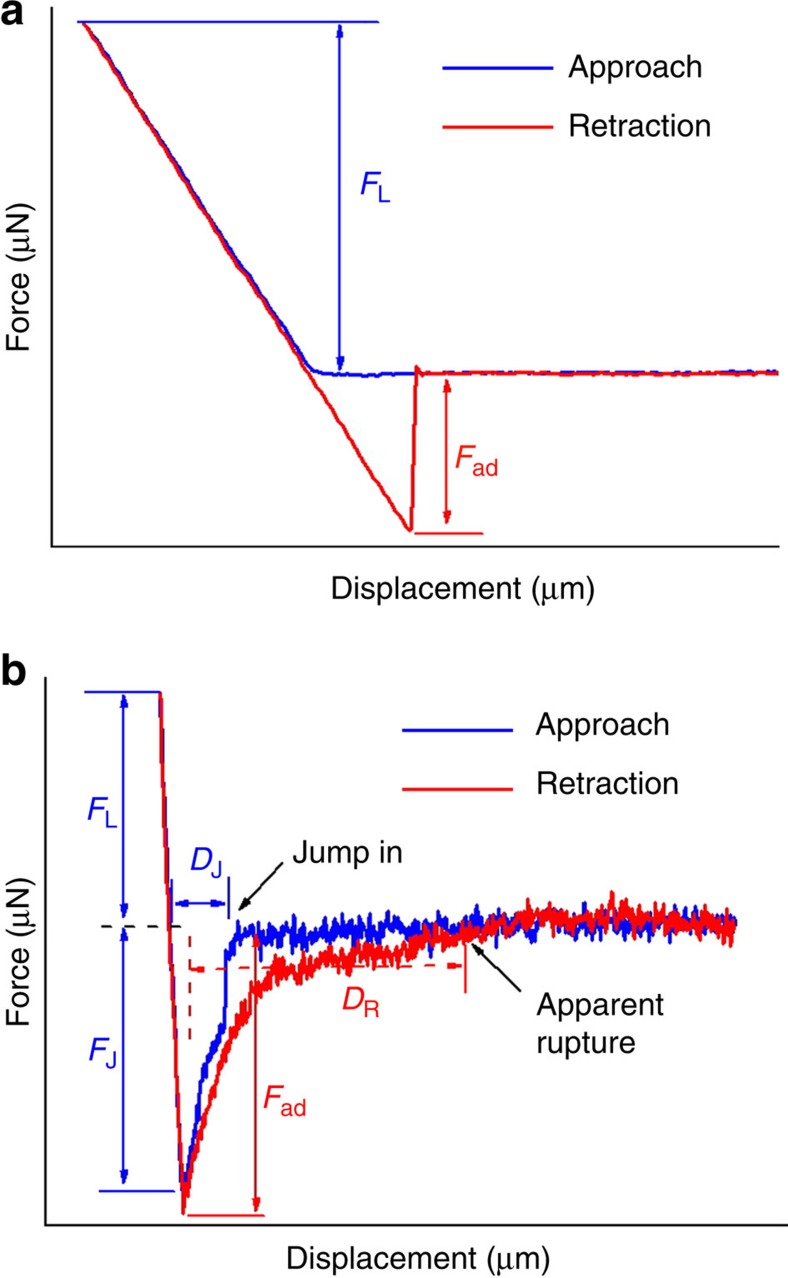
Comparison of force-displacement curves before and after mineral oil supply. (**a**) Force–distance curve representing a dry adhesion scenario measured on a PFAP in the absence of mineral oil. *F*_L_ is the loading force applied during approach, *F*_ad_ the pull-off force (or adhesion force) detected during retraction. (**b**) Force–distance curve measured on an oPFAP with a spherical sapphire probe covered with mineral oil before contact formation, which is characteristic of wet adhesion. Jump-in force *F*_J_, jump-in distance *D*_J_ and *F*_L_ characterizing the approach branch, as well as apparent rupture distance *D*_R_ and *F*_ad_ characterizing the retraction branch, are indicated.

**Figure 5:**
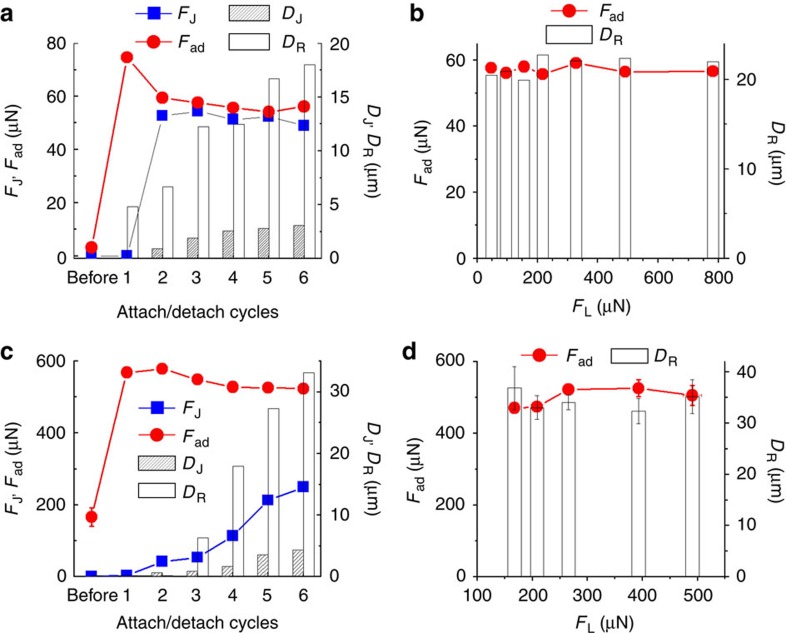
Evaluation of the force–distance curves. (**a**) Changes in *F*_ad_, *F*_J_, *D*_J_ and *D*_R_ in a series of force–distance curves successively measured at the same position at RH=25% with *F*_L_≈500 μN. ‘Before’ indicates mean values of *F*_ad_, *F*_J_, *D*_J_ and *D*_B_ obtained from three force–distance curves measured at different positions before the supply of mineral oil (error bars indicate s.d.). ‘1’ denotes the first and ‘2’ the second force–distance curve taken after the supply of mineral oil. Higher numbers denote further successively measured force–distance curves. (**b**) Dependence of *F*_ad_ and *D*_R_ on *F*_L_ obtained from a series of force–distance curves successively measured at the same position on an oPFAP at RH=25%. Before the measurements shown, the spherical sapphire was brought into contact with the tested oPFAP for 13 times at different positions. (**c**) Changes in *F*_ad_, *F*_J_, *D*_J_ and *D*_R_ in a series of force–distance curves successively measured at the same position at RH=90% with *F*_L_ ≈390μN. ‘Before’ and the numbers ‘1’ to ‘6’ have the same meaning as in the case of **a**. (**d**) Dependence of *F*_ad_ and *D*_R_ on *F*_L_ at RH=90%. Before the measurements shown, the spherical sapphire probe was brought into contact with the tested oPFAP for 10 times at different positions. Each data point in **d** represents at least three measurements at different positions of the tested oPFAP. s.d. of *D*_R_ and *F*_ad_ are indicated by the error bars.

**Figure 6:**
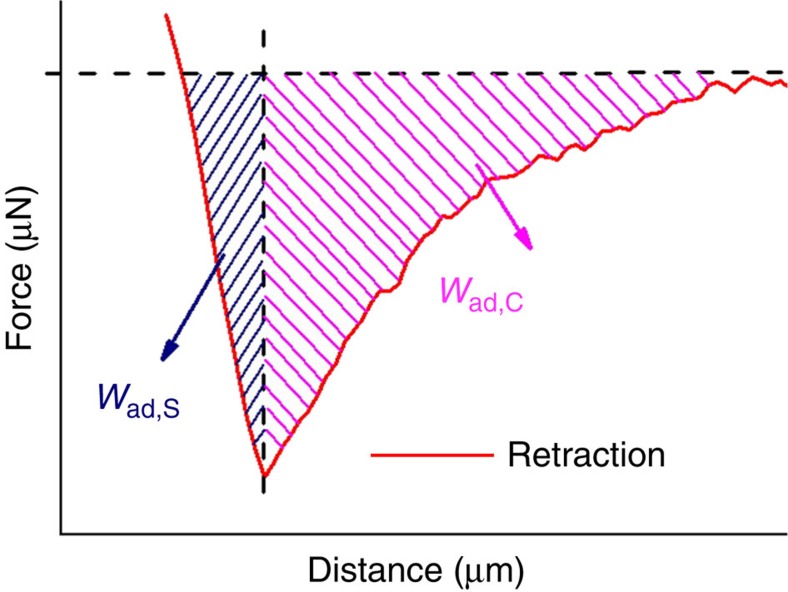
Work of adhesion required to separate oPFAPs from the counterpart surfaces. Shown is the retraction branch of a force–distance curve. The area between the intersection retraction branch/zero-force line and the displacement corresponding to force minimum *F*_ad_ (on the left; marked by dark blue lines) represents the work of adhesion *W*_ad,S_ required to break the solid–solid contact between oPFAP and the spherical sapphire probe. The area between the displacement corresponding to force minimum *F*_ad_ and the displacement beyond which the retraction branch coincides with the zero-force line (on the right; marked by lines in magenta) represents the capillary contribution *W*_ad,C_ to the work of adhesion, which is required to stretch and rupture liquid bridges between oPFAP and spherical sapphire probe.

**Figure 7:**
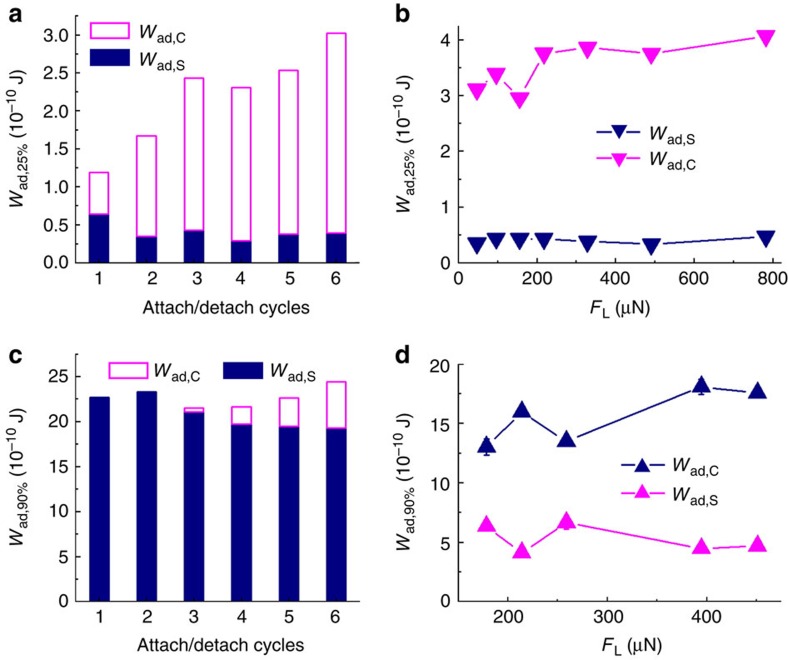
Work of adhesion on oPFAPs at RH=25% and RH=90%. (**a**) *W*_ad,S_ and *W*_ad,C_ determined from the series of force–distance curves evaluated in Fig. 5a, which were taken at the same position on an oPFAD at RH=25% with *F*_L_≈500 μN. ‘1’ denotes the first and ‘2’ the second force–distance curves taken after supply of mineral oil. Higher numbers denote further successively measured force–distance curves. (**b**) Dependence of *W*_ad,S_ and *W*_ad,C_ on *F*_L_ at RH=25% obtained from the series of force–distance curves, which were successively measured at the same position on an oPFAP and evaluated in Fig. 5b. Before the measurements shown, the spherical sapphire probe was brought into contact with the tested oPFAP for 13 times at different positions. (**c**) *W*_ad,S_ and *W*_ad,C_ determined from the series of force–distance curves, which were successively measured at the same position of an oPFAP at RH=90% with *F*_L_≈390 μN and evaluated in Fig. 5c.‘1’ denotes the first and ‘2’ the second force–distance curve taken after the supply of mineral oil. Higher numbers denote further successively measured force–distance curves. (**d**) Dependence of *W*_ad,S_ and *W*_ad,C_ on *F*_L_ at RH=90% determined from the set of force–distance curves evaluated in Fig. 5d. Each data point represents at least three measurements at different positions on the tested oPFAP. Before the measurements shown, the spherical sapphire probe was brought into contact with the tested oPFAP for 10 times at different positions.

**Figure 8:**
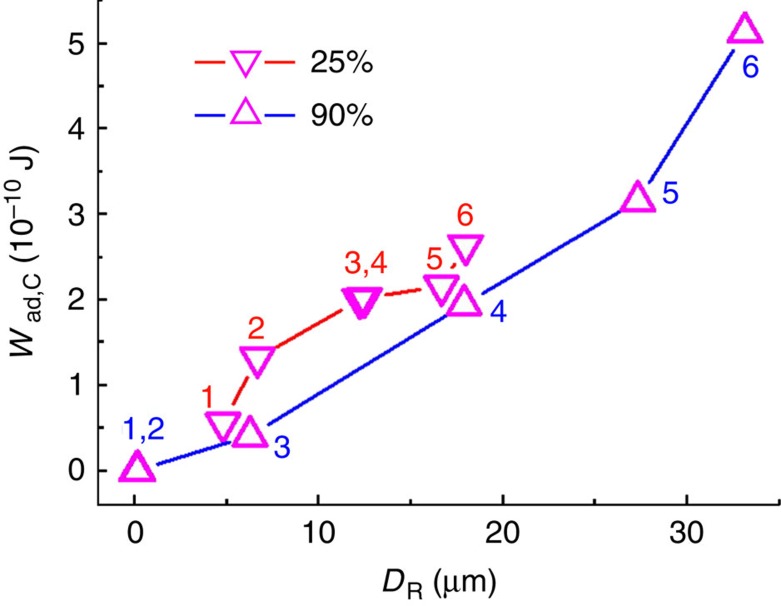
Dependence of *W*_ad,C_ at RH=25% and RH=90% on *D*_R_. Numbers 1–6 denote the first to the sixth attach/detach cycle performed after the supply of mineral oil.

The independence of *F*_ad_ on *F*_L_, which is in agreement with the classical Johnson−Kendall−Roberts theory^29^, as well as the strong increase in *F*_ad_ upon supply of mineral oil independent of RH may be rationalized by assuming capillarity-supported formation of solid-solid contact (Fig. 1). In the absence of mineral oil at the contact interface, the contact formation between the PFAP and the spherical sapphire probe is imperfect, as predicted by the modified SPG model. However, as an oPFAP is approached to a counterpart surface such as a spherical sapphire probe (Fig. 1a), liquid bridges form and exert attractive forces between the two solid surfaces connected by the liquid bridges (Fig. 1b). These attractive forces may be related to the surface tension of the liquid bridge (and the resulting capillary pressure) as well as to contact line tension. The tendency to form liquid bridges between the contact elements soaked with mineral oil and spherical sapphire probe tethers the contact elements to the spherical sapphire probe without the need of applying load (Fig. 1c). The enhanced solid–solid contact mediated by capillarity in turn increases *F*_ad_ and *W*_ad,S_.

oPFAPs may represent the starting point for the deliberate implementation of insect-inspired wet adhesion in the design of artificial adhesive systems. However, several features of oPFAPs may be further improved in the future. For example, we are not able to completely avoid the clustering of the fibrillar contact elements of oPFAPs. Enhancing the performance of oPFAPs by the prevention of clustering is a challenging optimization problem. On the one hand, clustering deteriorates the performance of fibrillar adhesive systems. On the other hand, straightforward changes in the array geometry that prevent clustering, such as the reduction of the contact elements’ aspect ratio or increasing in the nearest-neighbour distance between the contact elements, also inevitably reduce adhesion. We believe that hybrid systems consisting of contact elements of a stiffer material connected to compliant substrates may show even better performance than the oPFAPs tested in this work.

## Methods

### Materials

PS-*b*-P2VP (*M*_n_(PS)=101,000 g mol^−1^; *M*_n_(P2VP)=29,000 g mol^−1^; *M*_w_/*M*_n_ (PS-*b*-P2VP)=1.60) was obtained from Polymer Source Inc., Canada and used as received. Mineral oil, tetrahydrofuran and ethanol were purchased from Sigma-Aldrich.

### Microscopic characterization

SEM and cryo-SEM investigations were carried out on a Zeiss Auriga microscope operated at an accelerating voltage of 3 kV. The samples were sputter-coated with a 5 nm-thick iridium layer. White-light interferometry, an established non-contact surface profiling method^30^, was carried out using a NewView 6200 white-light interferometer (ZygoLOT GmbH, Darmstadt, Germany). The light reflection from the boundary between two materials with different refractive indexes is registered with the white-light interferometer. Because of the small difference in the refractive indexes, the reflection from the interface between oil (*n*=1.40) and the glass (*n*=1.47) is neglected compared with the reflection from the interface between oil and air (*n*=1.00).

### Adhesion tests

Force-displacement curves were measured at 20 °C on PFAPs and oPFAPs with areas of about 5 × 5 mm^2^ mounted on a hexapod nanopositioning stage (Physik Instrumente, Karlsruhe, Germany) using a home-built force tester Basalt-02. Sapphire spheres with a diameter of 3 mm glued to the free end of a metal spring with a spring constant *C*_spring_=69.8 N m^−1^ were used as probes that could be vertically moved with a piezo drive. The spherical sapphire probes were cleaned with acetone before a new series of measurements was carried out. The approach and retraction speeds of the spherical sapphire probes during the measurements were 2 μm s^−1^. Retraction started immediately when the predefined *F*_*L*_ values were reached. The deflection of the metal spring during the approach and the retraction was monitored with a laser interferometer and converted into force. For data collection, a custom-made Labview software package was used. All force-displacement curves were acquired in a homemade humidity chamber at room temperature, as described in detail in the supporting information to ref. 18. RH values were controlled by mixing dry N_2_ and saturated water vapour at different ratios.

## Author contributions

L.X., M.S. and S.N.G. designed the project. L.X. and A.E-V. fabricated the structure and measured with SEM. L.X. performed adhesion measurements and analysed the data. L.X. and M.S. wrote the paper. L.X., M.S., A.K. and S.N.G. discussed the results and commented on the paper.

## Additional information

**How to cite this article:** Xue L. *et al.* Humidity-enhanced wet adhesion on insect-inspired fibrillar adhesive pads. *Nat. Commun.* 6:6621 doi: 10.1038/ncomms7621 (2015).

## Supplementary Material

Supplementary Information Supplementary Figures 1-2, Supplementary Table 1 and Supplementary References
