# Supplementary material for: Humidity-enhanced wet adhesion on insect-inspired fibrillar adhesive pads
Source: Nat Commun. 2015 Mar 20;6:6621. doi: 10.1038/ncomms7621 (PMC4383020; doi:10.1038/ncomms7621)
Supplement: Supplementary Information — Supplementary Figures 1-2, Supplementary Table 1 and Supplementary References [file ncomms7621-s1.pdf]

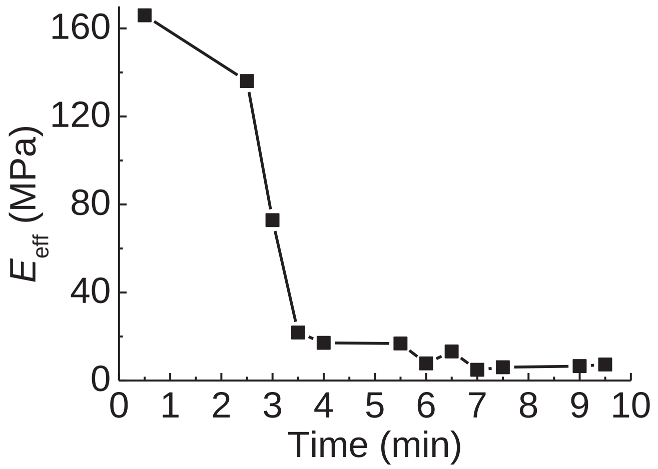

Supplementary Figure 1. Dependence of the Young modulus E_{eff} of a PFAP on the time during which N_2 with RH = 90% is piped into the sample chamber. [1]

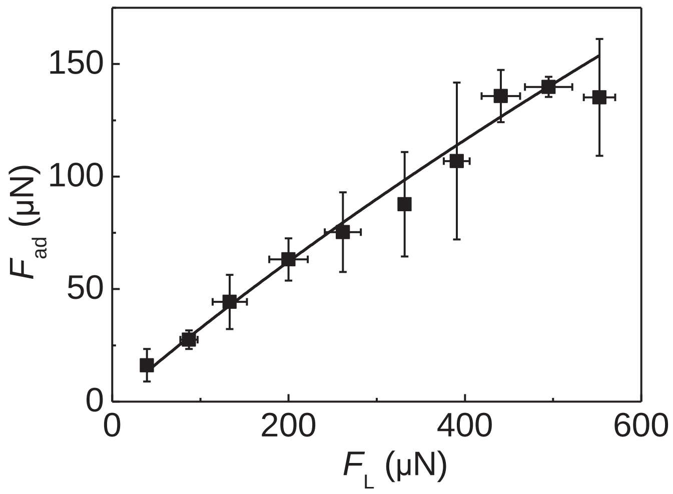

Supplementary Figure 2. Dependence of F_{ad} on F_L on a PFAP at RH = 90%. Each data point is the mean value of at least six adhesion measurements at different positions on two samples. The error bars indicate the standard deviations. The solid lines are fits obtained with the spring model described in reference [2].

Contact angle (º)	Poly(vinyl-2-pyridine)	Spherical sapphire probe
Water	65.8	85.3
Mineral oil	17.7	24.0

Supplementary Table 1. P2VP homopolymer film with a thickness of a few 10 µm (M_w = 41000 g/mol; M_n = 37000 g/mol; M_w/M_n = 1.11) was prepared by spin coating a solution of 10 wt-% P2VP in ethanol onto a silicon wafer at 1000 rpm, followed by drying in vacuum. The volumes of the liquid droplets (water and mineral oil) were 3 µL. The contact angle of mineral oil on a spherical sapphire probe was directly measured by placing the mineral oil droplet between the spherical sapphire probe and a glass slide.

Supplementary References

[1] Xue, L. *et al*. Reversible adhesion switching of porous fibrillar adhesive pads by humidity. *Nano Lett.* **13,** 5541−5548 (2013).

[2] Xue, L.*et al*. Tailoring normal adhesion of arrays of thermoplastic, spring-like polymer nanorods by shaping nanorod tips. *Langmuir* **28**, 10781−10788 (2012).
